# A quantitative comparison of devices for in vivo biomechanical characterization of human skin

**DOI:** 10.1007/s42558-023-00053-w

**Published:** 2023-07-17

**Authors:** Håvar J. Junker, Bettina Thumm, Sascha Halvachizadeh, Edoardo Mazza

**Affiliations:** 1 Present address: Institute for Mechanical Systems, Department of Mechanical and Process Engineering, ETH Zurich, Leonhardstrasse 21, Zurich, 8092 Switzerland; 2 Department of Trauma, University Hospital Zurich, Rämistrasse 100, Zurich, 8091 Switzerland; 3 Empa, Swiss Federal Laboratories for Materials Science and Technology, Überlandstrasse 129, Dübendorf, 8600 Switzerland

**Keywords:** Skin biomechanics, Myoton, SkinFibroMeter, Nimble

## Abstract

Non-invasive skin characterization devices are emerging as a valuable tool in clinical skin research. In recent years, the range of available experimental techniques and methods used to determine the biomechanical properties of skin has increased considerably. Although a substantial amount of work has been devoted to assessing the working principle of macroscopic skin characterization devices individually, a rationalization and comparison between them is still lacking. This motivated the present study, which aimed to characterize and compare three commonly used working principles: suction, dynamic shear loading, and indentation. A synthetic model system with tunable mechanical properties was used to assess the three devices, and the results rationalized based on corresponding finite element models. In vivo measurements were performed on healthy volunteers to investigate the capability of differentiating the biomechanical properties of skin at different body locations, and to assess the intra- and inter-rater reliability of each device. The present comparative analysis indicates that the analyzed functional principles perceive the stiffness of human skin differently, with relevant implications for the interpretation of the respective measurement results.

## Introduction

The skin is the largest organ of the human body, forming our primary protective barrier to the surrounding environment. From a mechanical perspective, skin is a highly complex material, capable of undergoing large deformations while displaying high fracture toughness as well as a capability of repairing injuries and growing [1–3]. On the microstructural scale, mechanical and biochemical cues go hand in hand to maintain the functional properties of the skin [4, 5]. Skin can be described as a multi-phasic soft-tissue, consisting of three main layers: epidermis, dermis, and hypodermis. Due to its heterogenous layered structure, pronounced tension-stretch nonlinearity, and time dependence associated with fluid flow and solid phase dissipation, skin remains a challenging material to characterize from a mechanical perspective [6–8].

The biomechanical properties of skin play a fundamental role in a wide range of medical applications and pathologies such as wound healing and the formation of scars, aging associated fragility as well as skin fibrosis [9–11]. Furthermore, recent advances in skin tissue engineering have highlighted the need for individually optimized properties of scaffolds, making their mechanical characterization with respect to native tissue highly relevant [12, 13]. However, conventional mechanical characterization techniques require excision of skin samples, making them unsuitable for other than research purposes.

More recently, non-invasive in vivo skin characterization devices have developed into promising tools for measurements at the tissue length scale. The functional principle behind these devices can vary greatly, not to mention the nature of the extracted parameters, pointing to a lack of standardization [14]. Only few attempts have been made to correlate and compare different devices, highlighting the need for a quantitative comparison of their ability to characterize the mechanical properties of skin [15]. This is the focus of the present study, addressing three commonly applied working principles: suction, dynamic shearing, and indentation.

First, measurements were performed with the different devices on elastomers with tunable mechanical properties in order to investigate the capability of each device to determine the substrate stiffness. Corresponding finite element (FE) simulations were performed to rationalize the measurements. In fact, correspondence between simulations and measurements indicated that the models reproduced the respective measurement principles well. Each device was then used to perform measurements on five human subjects in two different anatomical sites. The capability of distinguishing the two sites was assessed, as well as the intra- and inter-observer reliability through the means of the intra-class correlation coefficient (ICC) [16]. Based on the calibration with elastomers, corresponding apparent moduli of the measurements on volunteers were determined for each device, serving as a framework for directly comparing the different measurement principles.

## Methods

### Suction experiments

Suction experiments were performed with the Nimble, a skin suction device recently developed at ETH Zurich [17]. One of the key advantages of the Nimble is its lightweight design. Consequently, the device can be operated with small contact forces, greatly improving the reliability of the measurements [17]. It has been successfully applied in numerous preclinical and clinical studies, such as for assessing the severity of skin fibrosis and for longitudinal monitoring of scar maturation in pediatric burn patients [18–20].

**Fig. 1:**
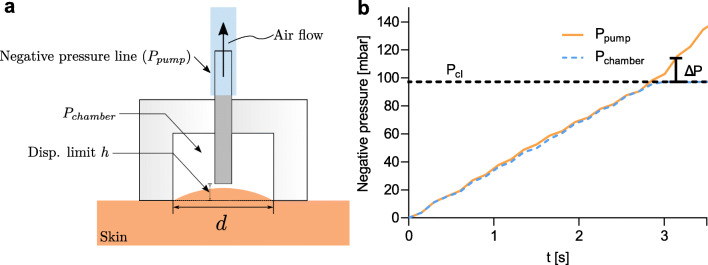
An overview of the principle of the suction device. **a** Sketch of the suction experiment with the skin in a deformed state. The air flow due to the negative pressure is indicated. **b** An example graph showing the output data from the device. The closing pressure *P*_*cl*_ is determined when the difference between the pump pressure *P*_*pump*_ (orange) and the chamber pressure *P*_*chamber*_ (dotted blue) reaches the pre-determined threshold Δ*P*

Its functional principle is based upon the application of a progressive negative pressure inside a cylindrical probe placed on the skin surface, together forming a closed chamber (Fig. 1 a). The measurement is considered to be quasi-static due to the slow pressure ramp applied (15 mbar s^− 1^). The negative pressure inside the chamber induces deformations in the skin layers. Eventually, the elevation of the skin reaches the position *h*, effectively sealing the suction pipe. This event is easily recognizable in the pressure-time diagram, as the pressure difference Δ*P* between the suction line and the chamber will rapidly increase when the suction pipe closes (Fig. 1 b). Consequently, the closing pressure *P*_*cl*_ can be extracted from the diagram when Δ*P* reaches a pre-defined threshold. The probe can easily be exchanged to accommodate different probe opening widths and tissue elevations. A probe diameter of *d* = 6 mm and a probe elevation of *h* = 1 mm was used in this study.

A corresponding FE model was constructed as an axisymmetric model in the FE software ABAQUS (Version 6.14, Dassault Systèmes Simulia, Johnston, USA). The suction was induced by a negative pressure boundary condition. The vertical displacement of the apex point was monitored, allowing the closing pressure to be extracted from the pressure-elevation diagram. The mesh was generated with a free meshing technique with a minimum seed size of 25 μm using axisymmetric elements with quadratic interpolation (*n* = 64,480). A figure showing the FE model boundary conditions and corresponding mesh can be found in the Appendix (Fig. 9).

### Dynamic shear force experiments

**Fig. 2:**
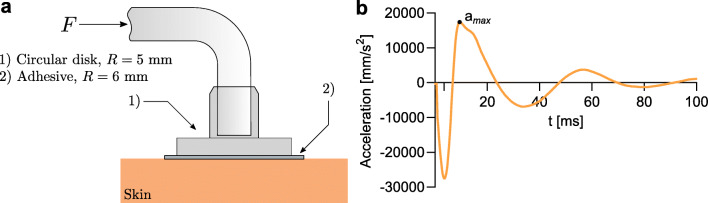
An overview of the principle of the dynamic shear force device. **a** Sketch of the shear probe attached to the skin. **b** An acceleration curve generated by the device used to calculate the dynamic stiffness. The parameter *a*_*max*_ needed for the calculation of the stiffness descriptor *S* has been indicated

Dynamic shear force experiments were performed with the MyotonPRO (Myoton AS, Tallinn, Estonia), a device originally developed to characterize the biomechanical properties of muscles and soft tissues [21, 22]. More recently, the mode of application was adapted to allow for shear force measurements on the skin surface [23]. A circular disk with a radius of 5 mm is adhered to the skin surface using double sided medical grade tape with a thickness of 0.09 mm (1577, 3M, Maplewood, USA). Thereafter, the handheld device is connected to the skin through an L-shaped probe (Fig. 2 a).

To initiate the measurement, a pre-force of 0.18 N is applied along the horizontal axis. Thereafter, a force impulse of 0.42 N (total load 0.60 N) is applied over an interval of 7 ms, during which the displacement and acceleration of the probe are recorded (Fig. 2 b). Ultimately, the stiffness parameter is extracted as:

1 $$ S = \frac{a_{max} \cdot m_{probe}}{\Delta u} \quad \Big[\frac{\text{ms}^{-2}\text{kg}}{\mathrm{m}}\Big] $$

where *a*_*max*_ represents the maximum acceleration (see Fig. 2 b), Δ*u* the maximum displacement from the position of the pre-tension phase, and *m*_*probe*_ the mass of the probe. The standard operating mode of the device outputs the average of five consecutive impulses.

The mirror symmetry about the probe axis was utilized when generating the FE model. The numerical analysis was performed using an explicit solver (ABAQUS Explicit, Version 6.14). The pre-force was applied quasi-statically to a reference point on the rigid disk to which the mass of 9 g was assigned. Thereafter, the impulse was implemented as a step function on the interval *t* ∈ [0 ms, 7 ms]. Dissipative behavior was not implemented in the model due to the substantial accompanying computational costs. The mesh was generated using three-dimensional tetrahedral elements with quadratic interpolation (*n* = 86,021) with a minimum seed size of 500 μm. An overview of the FE model boundary conditions and mesh can be found in the Appendix (Fig. 10).

### Indentation experiments

**Fig. 3:**
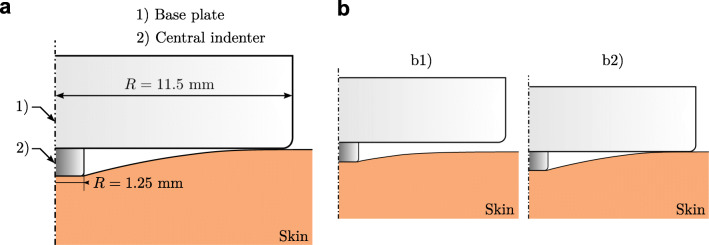
An overview of the working principle of the indentation device, shown in **a**. **b** The two stages of the indentation procedure have been depicted: b1) only the central indenter is in contact with the skin; b2) both the central indenter and base plate are in contact with the skin

Indentation experiments were performed with the SkinFibroMeter (SFM) (Delfin Technologies, Kuopio, Finland). It is a skin indentation device composed of two different force sensors: a base plate (*R* = 11.5 mm) and a central cylindrical indenter with a length and radius of 1.25 mm (Fig. 3 a). After indenting the skin, the force on the central indenter is reported when complete contact between the skin and base plate is detected (Fig. 3 b). The reported value is the mean value of five successive indentations, each of which lasting 0.5 s. The output of each single indentation is not accessible to the user.

The system was modelled exploiting the axisymmetric nature of the device. The mesh of the FE model was generated using axisymmetric triangular elements with quadratic interpolation (*n* = 23,379), with a minimum seed size of 100 μm. The indentation was realized by enforcing a displacement-controlled boundary condition of the base plate and central indenter, which were modelled as rigid bodies. The indentation force could then be extracted from the diagram showing the reaction force on the rigid bodies, where the event of full contact with the base plate is easily recognizable due to the sudden increase in force. An overview of the FE model boundary conditions and mesh can be found in the Appendix (Fig. 11).

### Synthetic material preparation and characterization

A model system with controllable mechanical properties was selected to evaluate the different devices. For this purpose, silicone elastomers were prepared, for which Young’s modulus can be controlled by varying the density of crosslinks. Two different elastomers were manufactured: Ecoflex (00-30, Smooth-On, Macungie, USA) mixed with a silicone thinner (weight ratio 1:1:1, Silicone Thinner, Smooth-On, Macungie, USA) (hereafter: EF) and polydimethylsiloxane (Sylgard 184, Dow, Midland, USA) with a base-to-crosslinker ratio of 35:1 ($\frac {w}{w}$) (hereafter: PDMS). After mixing the corresponding ratios, the mixtures were degassed in a vacuum desiccator before casting into petri dishes (PDMS: *D* = 89 mm, *m* = 60, g; EF: *D* = 87 mm, *m* = 45 g). The samples were then degassed again before curing (PDMS: 2 h at 80 ^∘^C; EF: 4 h at room temperature).

A third configuration was produced by spincoating (600 rpm, 90 s) a layer of the 35:1 PDMS on top of an already cured sample of a much softer PDMS with a base-to-crosslinker ratio of 45:1. The particular parameters for spincoating were chosen such that a layer thickness of about 100 μm was achieved. This configuration was used to investigate the capability of each device to detect the spincoated layer, when compared to measurements on the uncoated sample.

**Fig. 4:**
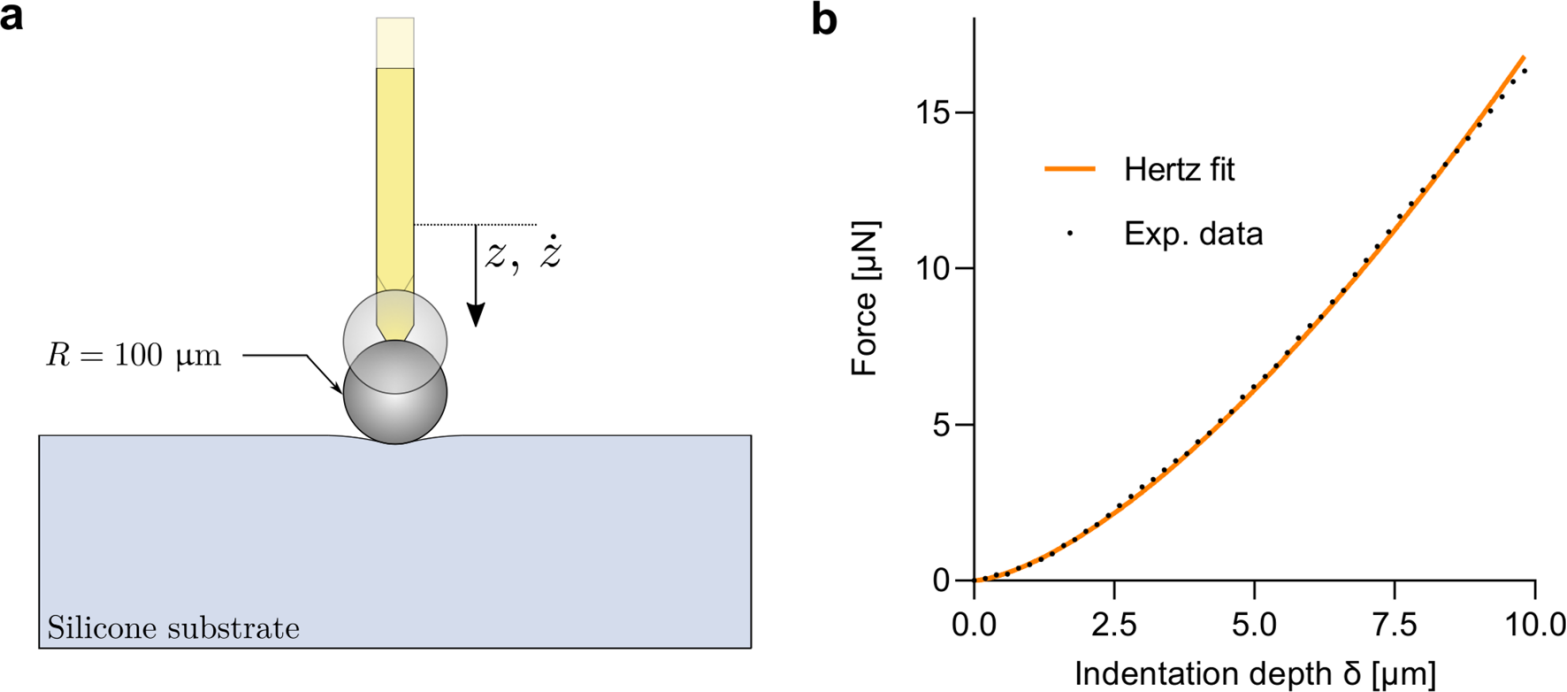
An overview of the microindentation procedure used for elastomer characterization. The procedure of the indentation experiment is shown in **a**, whereas the corresponding analytical fit of the experimental data is depicted in **b**

The stiffness of each material was measured by performing indentation experiments with a micromechanical testing rig (FT-MTA02, FemtoTools AG, Buchs, Switzerland) (Fig. 4 a). Force sensors with a range of ± 1000 μN and a sensitivity of 0.05 μN (FT-S1000, FemtoTools AG, Buchs, Switzerland) were modified by fixing high precision spheres with a radius of 100 μm (cubic zirconia, Sandoz Fils SA, Cugy, Switzerland) to the proximity of the sensors using UV light curing adhesive (AA3394, Henkel Loctite, Rocky Hill, USA). Force-displacement curves were acquired with a displacement rate of 1 μm s^− 1^ in steps of 0.2 μm. Each sample was indented in minimum 9 different locations separated by at least 500 μm. An apparent elastic modulus (*E*) was extracted from each force-displacement curve under the assumptions of the analytical Hertzian contact solution (Fig. 4 b). For a rigid, spherical indenter and a flat, elastic half-space, the relationship between the force on the indenter (*F*) and the elastic modulus of the material (*E*) is given by [24]:

2 $$ F = \frac{4}{3}\cdot \frac{E}{1-\nu^{2}}\sqrt{R}\delta^{3/2} $$

where *R*, *ν* and *δ* represent the radius of the indenting sphere, the Poisson’s ratio and the indentation depth, respectively. The elastomers were assumed to be incompressible, thus *ν* = 0.5 was used for the subsequent analysis. Previous work demonstrated a very good correspondence between Young’s modulus obtained from microindentation and from uniaxial tensile tests [25].

### Constitutive model

The elastomers were modelled as hyperelastic materials. For simplicity, the incompressible form of the neo-Hookean strain energy potential was used:

3 $$ \psi = c_{1}(I_{1}-3) $$

where $c_{1} = \frac {1}{2}\mu = \frac {1}{6}E$ (*μ*: shear modulus; *E*: Young’s modulus) and *I*_1_ = tr(**C**) represents the first invariant of the right Cauchy-Green deformation tensor **C**. The material model was validated by performing FE simulations of the aforementioned indentation experiments, which indicated excellent agreement between the experimental and simulated force-displacement curves. Note that a variety of different strain energy potentials have been proposed in literature for modeling the present silicone elastomers [26]. The simplistic model applied here was shown to be adequate for the numerical simulations of the three devices considered here.

### In vivo measurements

Five male volunteers (hereafter: VO1-VO5) in the age group 20–40 years were recruited at ETH Zurich in agreement with the ethical approval (ETH EK 2020-N-174). Each volunteer was examined in two different body locations by three different observers (O1–O3) with each device. Each observer performed four repeated measurements in each location. The two different locations, namely the volar forearm (VF) and the forehead over the temple (FH), were chosen based on their expected difference in skin stiffness, as reported in previous studies [17].

### Statistical analysis

The difference in stiffness between the two anatomical locations was investigated with an unpaired two-sided *t*-test for each volunteer using the mean value over the four repetitions from each observer (*n* = 3). The reliability was assessed through the means of the intraclass correlation coefficient (ICC). More specifically, the ICC(2,1) and ICC(2,k) (two-way random effects model, absolute agreement) were used to assess the single-rater and average inter-rater reliability in each location, respectively [27]. Likewise, the intra-rater reliability was determined using the ICC(2,1) (two-way mixed effects model, absolute agreement). The intra-rater reliability was calculated using each repetition for each observer. The categorization of ICC values was done according to the criterion developed by Cicchetti [28]: ICC ≤ 0.4: poor reliability, 0.4 <ICC ≤ 0.59: fair reliability, 0.6 ≤ ICC ≤ 0.74: good reliability, 0.75 ≤ ICC ≤ 1.0: excellent reliability. A correlational analysis was performed on each pair of devices. The degree of correlation was then assessed using the Pearson correlation coefficient and the corresponding *p*-value [29]. The statistical analysis was performed in R (RStudio, Boston, USA) and Prism (Version 9.1, Graphpad, San Diego, USA).

## Results

### Synthetic material characterization

**Fig. 5:**
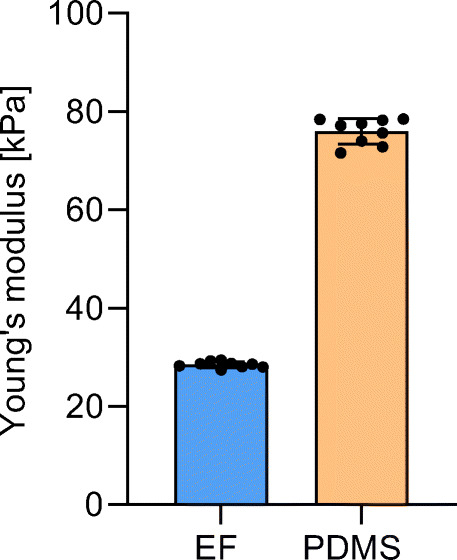
Overview of the stiffness values obtained with the microindentation tests on the elastomeric materials Ecoflex (EF) and polydimethylsiloxane (PDMS). Data are mean ± SD, individual data points shown with circles (*n* = 9)

The mean apparent modulus for the different elastomer configurations EF and PDMS was calculated from the corresponding force-displacement curves of the microindentation experiments (Fig. 5). These were found to be 28.5 kPa (coefficient of variance (CV): 2.17 %) and 76.0 kPa (CV: 3.43 %), respectively. This indicates a fold change of stiffness of 2.67 between the two elastomers. The minimum value of *R*^2^ was 0.99, indicating an excellent goodness-of-fit of the force-displacement curves.

### Suction experiments

#### Synthetic material characterization

**Fig. 6:**
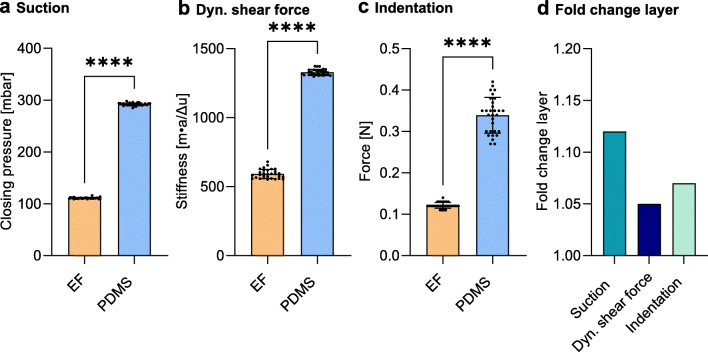
An overview of the results obtained on the synthetic model system using the different measurement techniques: **a** suction, **b** dynamic shear force, **c** indentation, and **d** the fold change of reported mean value between the layered configuration and bulk elastomer. EF: Ecoflex, PDMS: polydimethylsiloxane. Data are mean ± SD (*n* = 30). ns: *p* > 0.05; *: *p* < 0.05; **: *p* < 0.01; ***: *p* < 0.001; ****: *p* < 0.0001

The measurements performed on the synthetic model system with the suction device resulted in mean closing pressures of 111.0 mbar (CV: 1.34 %) and 292.0 mbar (CV: 0.93 %) for the EF and PDMS configuration, respectively, indicating a fold change of 2.63 (Fig. 6 a). By utilizing the material properties found in Subsection 3.1 as input values for the finite element analysis, the corresponding simulations yielded closing pressures of 115.7 mbar and 290.2 mbar, resulting in deviations of 4.2 % and 0.6 % with respect to the experiments. The fold change of the closing pressures found in the finite element analysis was calculated to be 2.51. With respect to the layered configuration, the suction device detected an increase of 12.2 % in the closing pressure relative to the bulk elastomer (Fig. 6 d).

#### In vivo measurements

**Fig. 7:**
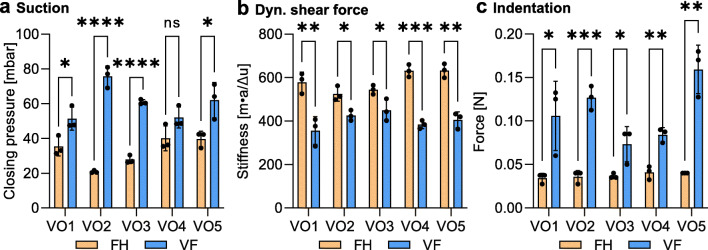
An overview of the measurements performed on the healthy volunteers (VO) using **a** suction, **b** dynamic shear force, and **c** indentation. *n* = 3 observers for each location (FH: forehead, VF: volar forearm) in each volunteer. Data are mean ± SD (*n* = 3). The difference between the two locations was statistically tested with a two-sided *t*-test. ns: *p* > 0.05; *: *p* < 0.05; **: *p* < 0.01; ***: *p* < 0.001; ****: *p* < 0.0001

The measured closing pressure in the FH and VF location is reported in Fig. 7 a for all volunteers. For each observer, the mean value of the four repetitions was used. The capability of detecting differences in stiffness between the two measurement locations was assessed for each volunteer through the means of a *t*-test. Significant differences were found in four out of five volunteers based on suction measurements. The mean closing pressure was found to be 32.8 mbar and 60.4 mbar in the FH and VF location, respectively, with corresponding ranges of 19.2 mbar and 24.3 mbar.

### Shear force experiments

#### Synthetic material characterization

The measurements on the synthetic model system using the dynamic shear force device resulted in mean values of 591.7 N m^− 1^ (CV: 5.64 %) and 1328.0 N m^− 1^ (CV: 1.62%) (see (1)) on the EF and PDMS configuration, respectively (Fig. 6 b). Note that a single impulse was used for each repetition on the elastomers. The fold change of stiffness between the EF and PDMS configuration was found to be 2.24.

The corresponding FE simulations for the EF and PDMS configurations indicated stiffness values of 648.4 N m^− 1^ and 1334.8 N m^− 1^, resulting in deviations of 9.6 % and 0.5 % to the experimental results. The fold change of stiffness between the two FE simulations was found to be 2.06. The measurements on the layered configuration resulted in an increase of 5.0 % in the stiffness relative to the bulk elastomer (Fig. 6 d).

#### In vivo measurements

The measurements on volunteers with the dynamic shear force device are reported in Fig. 7 b. Significant differences were found between the two locations for all volunteers. The mean stiffness value in the FH location was found to be 582.2 N m^− 1^ with a range of 107.8 N m^− 1^, whereas the mean in the VF location was found to be 404.4 N m^− 1^ with a range of 93.4 N m^− 1^.

### Indentation experiments

#### Synthetic material characterization

The measurements on the EF and PDMS resulted in mean force values of 0.122 N (CV: 5.75%) and 0.339 N (CV: 12.71%), respectively (Fig. 6 c). The fold change between the two bulk configurations was found to be 2.78. The FE calculations resulted in indentation forces of 0.133 N and 0.331 N for the EF and PDMS, respectively, indicating deviations of 9.0 % and 2.4 *%* to the experimental results. The fold change between the FE calculations was found to be 2.49. The measurements on the layered configuration resulted in an increase of 7.1 % in the indentation force relative to the bulk elastomer (Fig. 6 d).

#### In vivo measurements

Significant differences were found between the two locations for all volunteers (Fig. 7 c). The mean value for the FH location was found to be 0.038 N with a range of 0.007 N. The mean value for the VF location was found to be 0.110 N with a range of 0.086 N.

### Inter-rater reliability

The inter-rater reliability was assessed through the means of ICC(2,1) and ICC(2,k) (Table 1). The suction device provided high values, with values categorized as either good or excellent reliability. The shear force measurements were categorized as good and poor in terms of ICC(2,1), which improved to excellent and fair for ICC(2,k). For the indentation experiments, the measurement reliability in the VF location was categorized as good and excellent in terms of ICC(2,1) and ICC(2,k), respectively. However, the measurements performed on the FH resulted in an ICC(2,1) and ICC(2,k) value of 0, indicating that the device showed no capability of distinguishing the different volunteers in this location.

**Table 1:** Summary of inter-rater reliability in each measurement location (FH: forehead, VF: volar forearm), determined using ICC(2,1) and ICC(2,k)

		Functional principle		
	Location	Suction	Dyn. shear force	Indentation
ICC(2,1)	FH	0.74	0.65	0
	VF	0.65	0.32	0.64
ICC(2,k)	FH	0.89	0.85	0
	VF	0.85	0.58	0.84

### Intra-rater reliability

The intra-rater reliability was assessed using ICC(2,1). The average ICC(2,1) of all observers in each location has been reported in Table 2. The values obtained with the suction devices were both categorized as good, whereas those obtained with the dynamic shear force devices were categorized as good and fair. The values obtained from the indentation data were categorized as fair and excellent, respectively.

**Table 2:** Summary of the average intra-rater reliability values in each measurement location (FH: forehead, VF: volar forearm) quantified by ICC(2,1)

		Functional principle					
	Location	Suction		Dyn. shear force		Indentation	
ICC(2,1)	FH	0.63		0.71		0.48	
	VF	0.68		0.56		0.82	

The criterion used to categorize the values can be found in [28]

### Comparative analysis

**Fig. 8:**
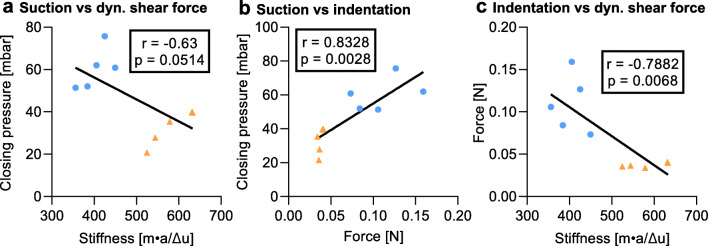
Overview of the correlation between measurement techniques: **a** suction vs dynamic shear force, **b** suction vs indentation, and **c** indentation vs dynamic shear force. The Pearson correlation coefficient (*r*) and corresponding *p*-value are indicated in each subfigure. The data points measured at the FH (forehead) location are shown as blue circles, whereas the data points measured at the VF (volar forearm) location are shown as orange triangles

**Fig. 9:**
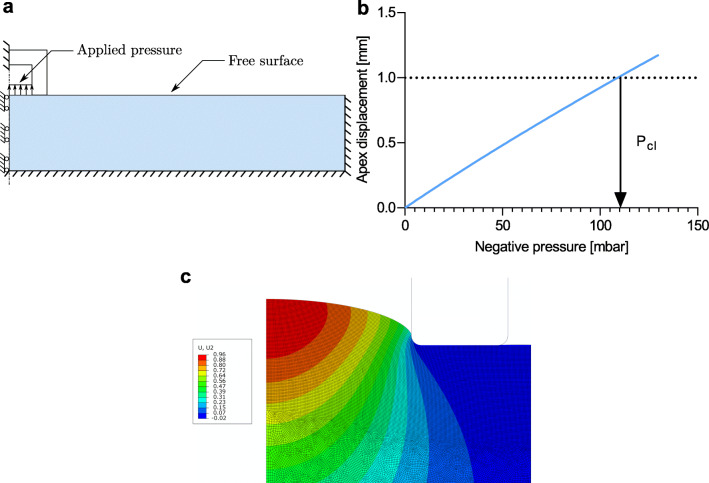
An overview of the FE model of the suction experiment. **a** A principal sketch of the finite element model where the BCs have been indicated. **b** The closing pressure is extracted by reading out the applied pressure when the apex displacement reaches the displacement limit *h*. **c** The mesh of the region around the suction chamber is shown. The color map indicates the vertical displacement of the elements

**Fig. 10:**
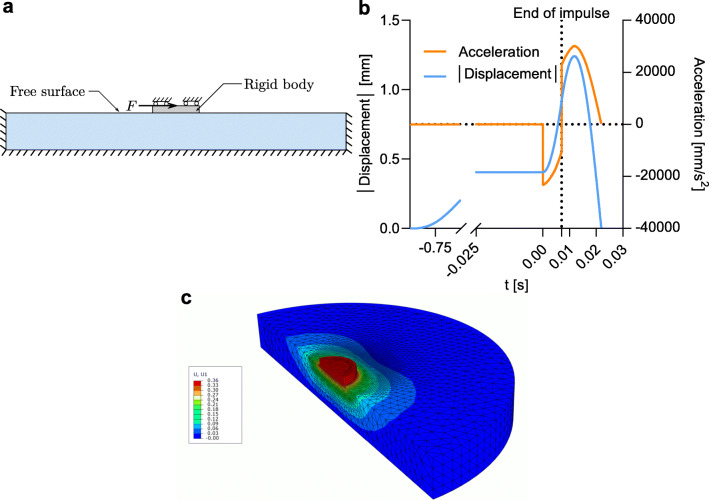
Overview of the FE model of the dynamic shear force experiment. **a** A principal sketch of the finite element model where the BCs have been indicated. **b** Curve of the acceleration and displacement of the rigid body generated with the model. Initially, the pre-force is applied gradually (note: The axis has been cut). Thereafter, the acceleration and displacement are monitored during and subsequent to the force impulse. **c** The mesh of the three-dimensional model, indicating the mirror symmetry used

**Fig. 11:**
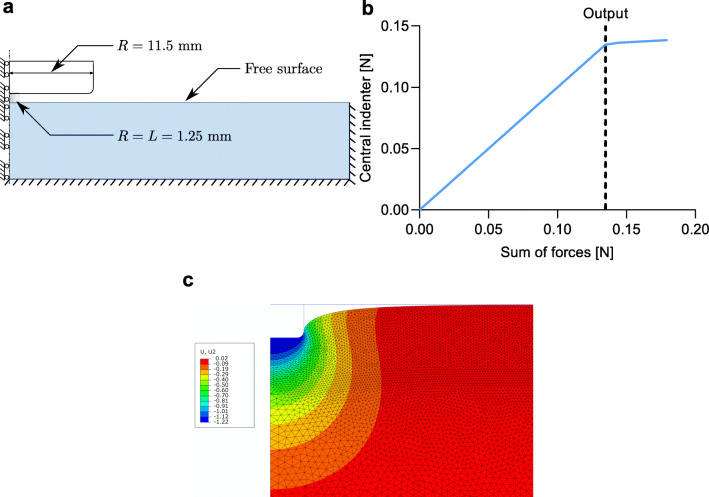
Overview of the FE model of the indentation experiment. **a** A principal sketch of the finite element model where the BCs have been indicated. **b** The indentation force is determined when complete contact with the base plate has been achieved. This can be observed when the slope in the force diagram changes, and the output value is read out (indicated in the figure). **c** The mesh of the region around the indenter is shown. The color map indicates the vertical displacement of the elements

To evaluate the correlation between the different devices, the Pearson correlation coefficient was calculated with the data from both locations for each combination of experimental techniques (Fig. 8) [29]. The suction experiments correlated strongly positively with the indentation experiments when considering the data points from both locations. Surprisingly, the shear force experiments correlated strongly negatively with the other two experimental techniques.

### Apparent modulus

Based upon the excellent agreement between the finite element model and experimental results on elastomers, an estimate of the perceived apparent modulus of each functional principle could be calculated from the in vivo measurements, where the mean of all measurements performed in each location was used as reference value (Table 3). For this purpose, the FE model used for the PDMS configuration was used for each device to compute an apparent modulus corresponding to the measured descriptor value. Notably, the suction and indentation model reported the same apparent modulus in FH location with a value of 8.6 kPa. Conversely, a similar agreement was not found in the VF location, where apparent moduli of 15.8 kPa and 25.2 kPa were obtained by the devices. The dynamic shear force measurements reported apparent moduli of 28.3 kPa and 17.6 kPa in the FH and VF location, with the largest discrepancy with respect to the other devices observed for measurements in the FH location.

**Table 3:** Summary of the apparent moduli corresponding to the measurements performed in vivo

	Functional principle					
	Suction		Dyn. shear force		Indentation	
Location	Descriptor (mbar)	App. mod. (kPa)	Descriptor (N/m)	App. mod. (kPa)	Descriptor (N)	App. mod. (kPa)
FH	32.8	8.6	582.2	28.3	0.038	8.6
VF	60.4	15.8	404.4	17.6	0.110	25.2

The mean of the pooled data in each measurement location (FH: forehead, VF: volar forearm) was used as descriptor value

## Discussion

In vivo skin characterization devices play an important role in clinical research given their capability of measuring skin properties non-invasively. However, the breadth of experimental techniques used in different devices lead to a wide range of extracted parameters and corresponding biomechanical properties. Motivated by the lack of unification and standardization between the different working principles of skin characterization devices, the present study aimed to compare three common skin characterization techniques: suction experiments using the Nimble, dynamic shear force using the MyotonPRO, and indentation experiments using the SkinFibroMeter.

### Synthetic material study

A preliminary study on synthetic materials was conducted in order to rationalize the different functional principles. The reference material properties of the synthetic materials were determined using high-precision micro-indentation equipment, which made it possible to observe to what extent the different devices could capture the corresponding values of material stiffness. These properties then served to calibrate a corresponding finite element model that was developed in parallel for each functional principle.

Overall, an excellent agreement was observed between the experimental results and corresponding numerical analysis for all three devices, with a maximal deviation of less than 10 %. The FE model of the dynamic shear force experiment did not account for rate dependent material behavior, which might be a contributing factor to the discrepancy between the computational and experimental results, as this device induces high shear rates. As shown herein, the measurements on the soft elastomer (EF) used in this study reported stiffness values comparable to healthy human skin. With stiffness values comparable to measurements on a matured scar, the stiff elastomer (PDMS) can be assumed to represent an upper bound of the physiological range of human skin stiffness [19]. Ultimately, all three devices were able to reliably distinguish the stiff-coated soft polymer from the uncoated configuration. This might indicate a similar capability of detecting microstructural changes in the skin.

### In vivo measurements

Considering that all devices reported an increased stiffness in the layered elastomer configuration under controlled boundary conditions, it is clear that the different functional principles lead to fundamentally different ways of perceiving the mechanical properties of skin. All three devices were able to successfully distinguish the two measurement locations in the in vivo measurements, noting that the suction device failed to detect a significant difference in one subject. Interestingly, the average relative range for the suction experiments was considerably higher than the other two functional principles. In contrast to the suction and indentation experiments, the shear force experiments showed a consistent decrease in stiffness between the forehead and volar forearm for all volunteers. The same effect was observed in a recent study done by Rosicka et al., where the skin stiffness was evaluated in different body locations using the MyotonPRO with indentation and shear probes, respectively [23]. Herein, an inconsistent variation in stiffness values between the different probes was reported, in line with the findings in this study. It is hypothesized that this effect occurs due to the influence from the level of deformation applied during the pre-tension phase of the measurement. This factor is expected to vary for each anatomical location, not only due to the properties of the underlying tissue, but also depending on the direction of imposed displacement, given the strong anisotropy of skin [30]. It should be noted that the MyotonPRO provides additional parameters that may contribute to a biomechanical characterization of skin. These parameters were not considered in the present study.

### Inter- and intraobserver variability

High repeatability and reliability are highly relevant for a skin characterization device to be considered for clinical use. In this study, suction experiments provided high values of inter-rater and intra-rater reliability. The inter-rater reliability was found to be zero for both measures (ICC(2,1) and ICC(2,k)) for the indentation experiments in the FH location. This indicates an incapability of distinguishing the different subjects. On the other hand, indentation provided the highest intra-rater reliability at VF, showing great repeatability. At FH, the intra-rater reliability was highest for the dynamic shear force device.

The comparative analysis indicated a positive intra-location correlation between the suction and indentation experiments, which supports the validity of both measurements.

### Apparent modulus

Motivated by the agreement between the experimental results and corresponding numerical simulations on the synthetic model system, an apparent modulus was calculated using the corresponding FE model for each measurement principle in each location. This analysis indicated a good agreement between the suction and indentation technique in the FH measurement site, whereas the indentation device reported a larger increase in stiffness value for the VF measurement site. This effect could be related to the influence of deeper layers at this location, increasing the apparent stiffness for compressive loads.

Notably, the dynamic shear force device operates at a deformation rate orders of magnitude higher than the other two devices used in this study, which can explain the different skin assessments. Furthermore, the neo-Hookean model applied in the present work represents a simplification not only of the tissue response but also for the elastomers considered in this study. A multilayer, time-dependent numerical model of the skin and the underlying structures could help explain the differences in measurement outcomes and facilitate the interpretation of the results from each device. In conclusion, it is evident that the reported apparent moduli can vary greatly between the different functional principles, so that a direct comparison of the stiffness descriptors is not possible.

## Conclusion

In this study, three different devices for in vivo skin characterization were analyzed and compared. Using synthetic materials and corresponding FE simulations, a framework for rationalizing the different functional principles was established. The subsequent analysis showed that the apparent moduli detected by the different devices on the synthetic materials were in excellent agreement. Moreover, all three devices were able to reliably detect a thin, stiff coating on a soft substrate. When performing measurements on healthy volunteers, the agreement between the devices was partially lost, as both the relative values and magnitude of the skin stiffness detected in each location differed between the devices. It is clear that the different devices respond differently to the complex structure of the skin at each anatomical location, which ultimately motivates a careful interpretation of the descriptors and the comparison between them.

## Data Availability

The datasets generated during the current study are available from the corresponding author on reasonable request.
